# Effects of skin care education for care staff at elderly care facilities on skin conditions of the residents

**DOI:** 10.1111/1346-8138.15213

**Published:** 2020-01-07

**Authors:** Yuichiro Tsunemi, Gojiro Nakagami, Kimie Takehara, Nao Tamai, Aya Kitamura, Yuko Mugita, Makoto Oe, Momoyo Kishida, Hiromi Sanada

**Affiliations:** ^1^ Department of Dermatology Saitama Medical University Saitama Japan; ^2^ Department of Gerontological Nursing/Wound Care Management Graduate School of Medicine The University of Tokyo Tokyo Japan; ^3^ Global Nursing Research Center Graduate School of Medicine The University of Tokyo Tokyo Japan; ^4^ Department of Nursing Graduate School of Medicine Nagoya University Nagoya Japan; ^5^ Department of Imaging Nursing Science Graduate School of Medicine The University of Tokyo Tokyo Japan; ^6^ Department of Medical Affairs Maruho Co., Ltd Osaka Japan

**Keywords:** asteatosis, care staff, education intervention, elderly care facilities, skin care

## Abstract

Asteatosis is common in elderly people due to a decrease in the moisture content of the epidermal stratum corneum through a loss of skin barrier function caused by aging. Because itching often accompanies asteatosis, this condition may cause a decrease in quality of life. Care staff in elderly care facilities have many opportunities to provide care for residents. In this study, we examined how educational training on skin care changed the thoughts and actions of care staff in these facilities and how these changes impacted the skin conditions of residents. The subjects for the training were all care staff in facilities because these staff work most closely with facility residents. We performed skin care training for the subjects and investigated changes in the skin conditions of the residents before and after the training. The training promoted the understanding of skin care among the care staff and improved the skin symptoms of residents with asteatosis. However, there were no changes in the severity of itchiness based on a verbal rating scale and in interviews of residents. This study showed that skin care training for the care staff in facilities is effective to improve skin conditions of residents. In addition, it was suggested that a full grasp of the residents’ skin symptoms based upon an interview on itching alone was difficult, and thus there is a need to observe skin conditions directly.

## Introduction

Asteatosis is a common in condition among elderly people in which the skin becomes dry due to a decrease of moisture content in the epidermal stratum corneum and reduced skin barrier function.^1^ Asteatosis decreases quality of life (QOL) due to itching. Asteatosis can lead to asteatotic eczema with various stimuli from the environment and scratches, inducing intense itchiness. Therefore, appropriate skin care is needed for asteatosis. Moisturizing agents can improve dry symptoms. Because many elderly people may not be aware of the development of asteatosis, caregivers need to notice their symptoms.

In a questionnaire survey of physicians engaged in medical care at elderly care facilities,^2^ the rate of asteatosis noted by the physicians (37.0%) and the use of moisturizing agents (39.7%) were similar, but much lower than the rate of asteatosis found by dermatologists (94.1%).^3^ In addition, this survey showed that most of the nurses did not correctly recognize asteatosis and asteatotic eczema in the residents.^3^ These findings suggest that skin care is not provided adequately to elderly residents in care facilities due to insufficient understanding and recognition of asteatosis among the staff.

In a previous study, we examined the effects of educational training to promote understanding about dry skin and its treatment among nurses and care staff (caregivers other than nurses) at elderly care facilities.^4^ The level of understanding of the use of moisturizing agents was improved by the training, and the frequency of observation of skin conditions of the residents and that of questions about the presence and level of dry skin and itching increased. Furthermore, the nurses recognized increased interest among care staff in the skin of the residents, and cooperation between physicians, nurses and care staff was improved. However, the effects of changes in the understanding and actions of care staff on skin conditions of the residents were not examined in the previous study. In this study, therefore, we performed similar training in other elderly facilities and investigated changes in the skin conditions of the residents before and after the training.

## Methods

### Study system

This study was performed mainly by the Non‐Profit Organization (NPO) Health Institute for Skin Research. The secretariat work was commissioned to EBC&M LLC (EBC&M). Severity of dry skin, scratch mark score and adverse events among the residents were evaluated by a dermatologist (investigator), and other efficacy evaluations were performed by nurses (researchers). Statistical analyses were performed at EBC&M. Monitoring of the research facilities was performed by Maruho (Osaka, Japan). The audit for implementation and management of monitoring was performed by a person who was appointed by the investigator.

This study was performed in accordance with the Declaration of Helsinki, the Ethical Guidelines for Medical and Health Research Involving Human Subjects (22 December 2014, Ministry of Education, Culture, Sports, Science and Technology, Ministry of Health, Labor and Welfare), and Ethical Guidelines for Medical and Health Research Involving Human Subjects (9 February 2015), after review and approval by the ethics committee of the medical corporation Shinkokai group (IRB no. 15000222). The study is registered in the Clinical Trials Registry of the University Hospital Medical Information Network (ID: UMIN000028881). The study was performed from October 2017 to May 2018 in four assisted‐living or housing‐type pay nursing homes operated by Benesse Style Care in Tokyo and Saitama Prefecture.

### Study design

**Figure 1:**
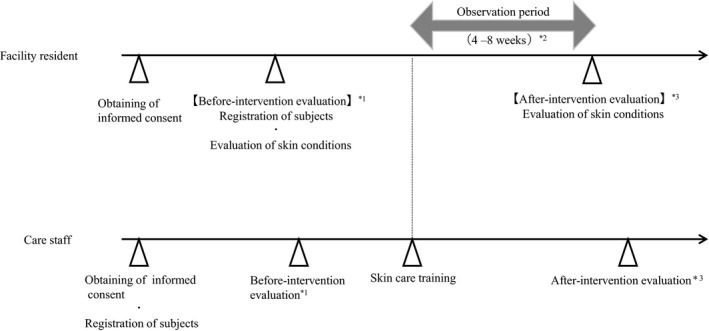
Study design. *1: The before intervention questionnaire survey of care staffs was performed on the same day or later than the before intervention evaluation of facility residents. *2: If a care staff member wished to additionally use a moisturizing agent for a resident during the observation period, the moisturizing agent prescribed by a physician who treated the resident was used. *3: The after intervention questionnaire survey of care staffs was performed on the on the same day or later than the after intervention evaluation for facility residents.

The study was performed as an open‐label controlled before–after intervention study. The study design is shown in Figure 1. Care staff (caregivers other than nurses) who worked at the elderly care facilities in the study were provided with skin care educational training on asteatosis and skin care. Before–after intervention questionnaire surveys were used to evaluate changes in the thoughts and actions of the staff. The skin conditions of facility residents were evaluated before the skin care training and after an observation period of 4–8 weeks after the training. Moisturizing agents were used for the resident if a care staff member felt the need for moisturizers during the observation period. The skin care training included explanations of the clinical conditions, symptoms and ways to deal with the symptoms of asteatosis and asteatotic eczema. Appropriate methods for the application of a moisturizing agent were explained using the “fingertip unit”^5^ as a rough standard for the amount to be applied. The training was provided for approximately 1 h.

### Inclusion and exclusion criteria

The subjects for the training were all care staff because these staff work most closely with facility residents. Each care staff member provided written informed consent after an investigator provided an explanation of the study details. No inclusion and exclusion criteria were used for the care staff. Among the residents, those with asteatosis in the lower extremities who were judged in the before–intervention evaluation to be unlikely to have major changes in their general condition throughout the study period were selected as subjects from among those who provided written informed consent. Residents who had skin disease over a wide area, inflammatory skin disease of a lower extremity, a history of allergy to moisturizing agents for external use, had participated in another clinical study within 4 months before the start of this study or were judged to be inappropriate as subjects of the study by the investigator were excluded.

### Observations and end‐points

#### Background of residents

Information on sex and age were obtained in the before–intervention evaluation.

#### Severity of dry skin

In the before–after intervention evaluations, a dermatologist evaluated the severity of dry skin on the forearms, lower extremities and trunk (chest/back) on a 5‐point scale based on the overall dry skin score: 0, absent; 1, faint scaling, faint roughness and dull appearance; 2, small scales in combination with a few larger scales, slight roughness and whitish appearance; 3, small and larger scales uniformly distributed, definite roughness, possible slight redness and a few superficial cracks; and 4, dominated by large scales, advanced roughness, redness present, eczematous changes and cracks.^6^

#### Severity of itching (subjective symptom)

Residents evaluated their severity of generalized itchiness in the 24 h before the before–after intervention evaluations on a 4‐point verbal rating scale (VRS) developed by Phan *et al*.: 0, no itch; 1, low; 2, moderate; and 3, severe itch.^7^

#### Moisture content in the epidermal stratum corneum and transepidermal water loss (TEWL)

These data were measured before–after intervention using a Corneometer and a Tewameter, respectively (both from Courage + Khazaka electronic, Koln, Germany). The measurements were performed at one site (3 cm × 3 cm in area) of a lower extremity and the mean of five independent measurements in each evaluation was taken. The room temperature and relative humidity shown by the device at the start of measurement were recorded for each evaluation.

#### Scratch marks

A dermatologist evaluated the presence of scratch marks at observation sites on a 4‐point scale: 0, none; 1, a few; 2, many; and 3, an extremely large number.

#### Interview of residents on subjective skin symptoms

In each evaluation, the residents were asked to describe their skin conditions according to the questionnaire in Table 1.

**Table 1:** Items in the before–after intervention interviews of facility residents

PQ1	Do you think your skin is rough (dry)?
Answer options	Agree/ Agree somewhat/No opinion/Disagree somewhat/ Disagree
PQ2	What do you do for your skin when you get worried about your rough skin?
Answer options	Using skin cream or lotion on my own/Consult with family/Consult with facility staff/Do nothing/No experience in worrying about rough skin
PQ3	Do you worry about itchiness of your skin?
Answer options	Frequently/Sometimes/Never
PQ4	Do you have sleepless nights due to skin conditions (e.g. itchiness)?
Answer options	Frequently/Sometimes/Never
PQ5	Have you received an explanation of treatment of rough skin from the care staff?
Answer options	Frequently/Sometimes/Never/Do not remember
PQ6	Do you feel that the care staff carefully observed your skin conditions?
Answer options	Agree/Agree somewhat/No opinion/Disagree somewhat/Disagree
PQ7	What kind of changes did you experience in the level of rough skin during the last 1 month?
Answer options	Gets better/Gets better somewhat/No change/Gets worse somewhat/Gets worse
PQ8	What kind of changes did you experience in the level of itchiness during the last 1 month?
Answer options	Gets worse/No change/Gets better/Do not remember
PQ9	What kind of changes did you experience in the frequency of sleepless nights due to skin conditions (e.g. itchiness) during the last 1 month?
Answer options	Increased/No change/Decreased/Do not remember

PQ1–PQ6, patient questions performed before and after the training; PQ7–PQ9, patient questions performed only after the training.

#### Adverse events

Information on adverse events in residents during the study period were collected for safety analysis. Adverse events included all unfavorable or unintended symptoms, diseases and aggravation of present diseases. A causal relationship with the study was not taken into consideration.

#### Questionnaire survey for care staff

A questionnaire survey for care staff on their thoughts and actions related to skin care was administered before and after the training session using the questionnaire in Table 2.

**Table 2:** Items on the questionnaire survey for the care staff (after training)

SQ1	How do you recognize dry skin or itchiness of facility residents? (multiple answers)
Answer options	Information from resident/Family/Physician/Nurse/Other care staff/Pharmacist/Myself/Others
SQ2	How much do you understand the methods to apply a moisturizing agent (type, application site, way to apply, amount and frequency)?
Answer options	Understand sufficiently to instruct/Almost understand/Understand insufficiently/Do not understand at all
SQ3	What did you instruct facility residents regarding the methods to apply a moisturizing agent? (multiple answers)
Answer options	Type/Application site/How to apply/Amount/Frequency/No instruction
SQ4	Did the number of observations of skin conditions in facility residents change, compared with that before the training?
Answer options	Increased/No change/Decreased
SQ5	Did the number of opportunities to ask the facility residents about their dry skin, presence and severity of itchiness change compared with that before the training?
Answer options	Increased/No change/Decreased
SQ6	Did the number of instructions on methods to apply moisturizing agents to facility residents (type, application site/how to apply/amount/frequency) change compared with those before the training?
Answer options	Increased/No change/Decreased
SQ7	Did the number of instructions on methods to apply moisturizing agents to other care staff (type, application site/how to apply/amount/frequency) change compared with those before the training?
Answer options	Increased/No change/Decreased
SQ8	Did the amount of moisturizing agents used for facility residents change compared with that before the training?
Answer options	Increased/No change/Decreased
SQ9	Did the number of applications of moisturizing agents to facility residents change compared with that before the training?
Answer options	Increased/No change/Decreased
SQ10	Did the rate of transfer of the need to use a moisturizing agent for a facility resident to a nurse change compared with that before the training?
Answer options	Increased/No change/Decreased
SQ11	Did the correspondence to a message transferred from a nurse about the skin care change compared with that before the training?
Answer options	Opportunities to forget the transfer: Decreased/No change/Increased
SQ12	Did the time required for you to deal with skin problems in facility residents change, compared with that before the training?
Answer options	Decreased/No change/Increased

SQ, staff question.

### Statistical analysis

#### Efficacy analysis

Residents who fell into any of the following categories were excluded from the efficacy analysis: no asteatosis in a lower extremity; no data for efficacy in the before–after intervention evaluations; not meeting the inclusion criteria; meeting an exclusion criterion; failure to follow directions on each of the evaluation days; and protocol violation. All other residents were included in the group for efficacy analysis.

#### Safety analysis

All residents were included in the safety analysis, except for those who had no data on safety in the before intervention evaluation or later.

#### Questionnaire survey analysis

All care staff at each facility were potential subjects, but those without data on the before–after intervention questionnaires were excluded. Analysis was performed by preparing a cross‐tabulation of the before–after intervention survey results.

#### Primary end‐point

The overall mean dry skin scores of the residents between the before–after intervention evaluations were compared by paired Wilcoxon test.

#### Secondary end‐points

In evaluation of the before–after intervention results, the mean ± standard deviation (SD) of itching VRS and scores for scratch marks were compared by paired Wilcoxon test, and the mean ± SD of moisture content in the epidermal stratum corneum and TEWL were compared by paired *t*‐test. To check the measurement condition on each evaluation day, the mean ± SD of room temperature and relative humidity were also compared by paired *t*‐test. In the interview of residents regarding subjective skin symptoms, cross‐tabulation was performed using the before–after intervention responses to the questions. For the same question in each evaluation, a χ^2^‐test was used to evaluate the response rates. The answers were also categorized to examine the responses in each evaluation.

All tests were two‐sided with a significance level of 5%. No supplements were provided for missing values.

## Results

### Composition of the subjects

The subjects included 28 facility residents (two men and 26 women) who provided informed consent (registered residents), of whom 28 were included in the safety analysis and 24 in the efficacy analysis, after exclusion of two subjects who discontinued participation in the study and two who deviated from the evaluation directions. The age (mean ± SD) of the subjects was 85.9 ± 5.7 years. A total of 22 care staff participated in the study, and 19 were included in the analysis, after exclusion of three subjects with missing data.

### Severity of dry skin

**Figure 2:**
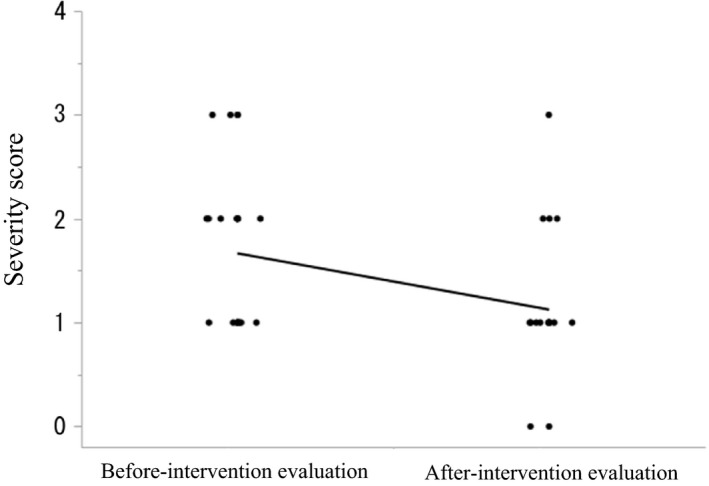
Severity of dry skin in lower extremities. Severity score: 0, absent; 1, faint scaling; faint roughness and dull appearance; 2, small scales in combination with a few larger scales, slight roughness and whitish appearance; 3, small and larger scales uniformly distributed, definite roughness, possibly slight redness and possibly a few superficial cracks; and 4, dominated by large scales, advanced roughness, redness present, eczematous changes and cracks.

The mean ± SD severity scores did not differ significantly in the before versus after intervention evaluations for the forearm (0.83 ± 0.56 vs 0.58 ± 0.50, *P* = 0.06), front of the trunk (0.71 ± 0.75 vs 0.71 ± 0.55, *P* = 0.81) and back of the trunk (0.88 ± 0.54 vs 0.83 ± 0.56, *P* = 0.71), but the after intervention score for the lower extremities was significantly lower (1.67 ± 0.76 vs 1.13 ± 0.61, *P* = 0.0006). These data are presented in Figure 2.

### Severity of itching (subjective symptoms)

There was no significant difference in VRS score in the before versus after intervention evaluations (0.6 ± 1.1 vs 0.6 ± 1.0, *P* = 0.59). The incidences of subjects with a VRS of 0 were 70.0% (16/23) and 73.9% (17/23) in the respective evaluations.

### Moisture content in the epidermal stratum corneum and TEWL

**Figure 3:**
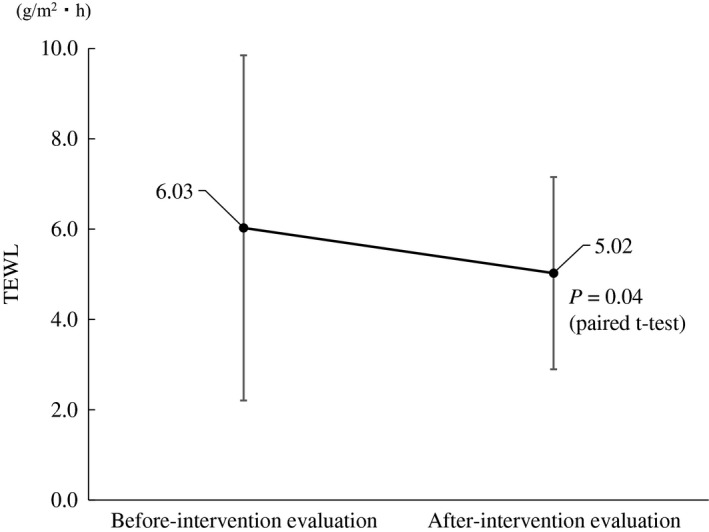
Transepidermal water loss (TEWL) in lower extremities.

In the after intervention evaluation, the mean moisture content in the epidermal stratum corneum was higher, but the difference was not significant (29.58 ± 7.47 vs 32.22 ± 7.87 arbitrary units, *P* = 0.10); the mean TEWL was decreased significantly (6.03 ± 3.82 vs 5.02 ± 2.13 g/m^2^·h, *P* = 0.04, Fig. 3). The room temperature was 24.4 ± 1.4 and 24.1 ± 0.9°C (*P* = 0.28) and the relative humidity (RH) was 32.6 ± 6.8 and 31.5 ± 7.3% RH (*P* = 0.51) for the before and after intervention evaluations.

### Scratch marks

There were no significant differences in the scratch mark scores in the before versus after intervention evaluations for the forearm (0.08 ± 0.28 vs 0.13 ± 0.34, *P* = 0.57), front of the trunk (0.08 ± 0.28 vs 0.00 ± 0.00, *P* = 0.16) and back of the trunk (0.29 ± 0.55 vs 0.13 ± 0.34, *P* = 0.08), but the mean scratch mark score for the lower extremities was significantly lower after the skin care training (0.29 ± 0.55 vs 0.04 ± 0.20, *P* = 0.02).

### Interview on subjective skin symptoms

**Figure 4:**
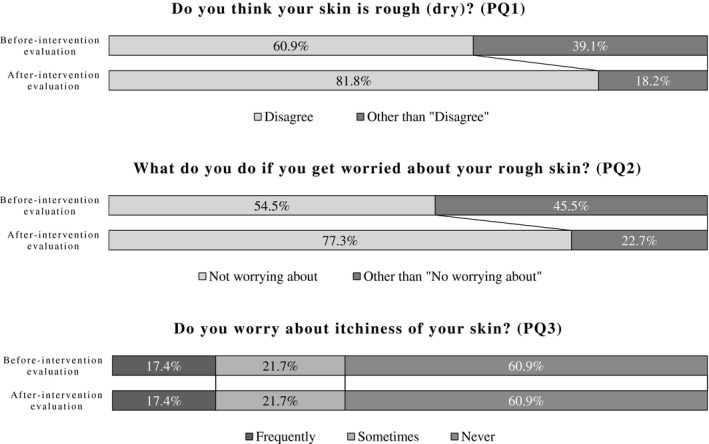
Interview of facility residents on skin conditions. “Disagree” includes patient question (PQ)1 answer “Disagree somewhat” and “Disagree”. Other than “Disagree” includes PQ1 answer “Agree” and “Agree somewhat”. “Not worrying about” includes PQ2 answer “No experience in worrying about rough skin”. Other than “Not worrying about” includes PQ2 answer, “Using skin cream or lotion on my own”, “Consult with family” and “Consult with facility staff”.

Data are reported for the before versus after intervention evaluations. For patient question (PQ)1, “Do you think your skin is rough (dry)?”, the ratio of being categorized as “Disagree” that includes the answers “Disagree somewhat” and “Disagree” showed a tendency to increase (60.9% vs 81.8%, *P* = 0.12). For PQ2, “What do you do for your skin when you get worried about your rough skin?”, the incidence of being categorized as “Not worrying about” that did not have experience of worrying about rough skin showed a tendency to increase (54.5% vs 77.3%, *P* = 0.11). However, for PQ3, “Do you worry about itchiness of your skin?”, there was no difference between the before and after intervention evaluations. Figure 4 shows the rates of the categorized answers for questions PQ1, PQ2 and PQ3.

For PQ7, “What kind of changes did you experience in the level of rough skin in the last 1 month?”, 90.9% answered that they had no change. Regarding itchiness, for PQ4, “Do you have sleepless nights due to skin conditions (e.g. itchiness)?”, the rates for “Never” were 87.0% and 90.9% in the before and after intervention evaluations, respectively. For itchiness in the last 1 month, “No change” had rates of 86.4% for PQ8, “What kind of changes did you experience for itchiness?”, and 90.9% for PQ9, “What kind of changes did you experience in the frequency of sleepless nights due to skin conditions (e.g. itchiness) during the last 1 month?”. No tendencies were evident for questions on support from care staff, including PQ5, “Have you received an explanation of treatment of rough skin from the care staff?”, and PQ6, “Do you feel that the care staff carefully observed your skin conditions?”.

### Safety evaluation

There were seven adverse events in five subjects, with an incidence of adverse events of 17.9% among the 28 subjects. All events occurred at sites other than the skin, and none of them were related to drugs used for asteatosis.

### Questionnaire survey for care staff

Data are reported for before versus after intervention. For question staff question (SQ)1, “How do you recognize dry skin or itchiness of facility residents?”, the answer “Nurse” increased after the training (36.8% vs 63.2%, *P* = 0.76). Similarly, for SQ3, “What did you instruct facility residents regarding the methods to apply a moisturizing agent?”, the answer application “Amount” increased after the training (15.8% vs 36.8%, *P* = 0.36). For SQ2, “How much do you understand the methods to apply a moisturizing agent?”, there was significant change (*P* = 0.003, χ^2^‐test) in the answers “Understand sufficiently to instruct” (0.0% vs 10.5%) and “Almost understand” (31.6% vs 78.9%).

**Figure 5:**
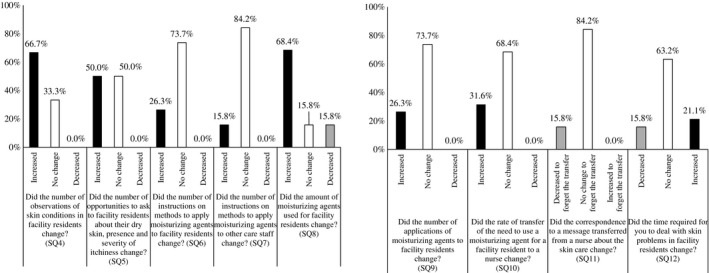
Questionnaire survey for care staffs (after the training). All changes were compared with that before the training.

The responses from the care staff after the training are shown in Figure 5. Although 66.7% of the care staff responded that their observation of skin conditions of facility residents increased, compared with that before the training (SQ4), the number of staff who responded that the opportunities to ask residents about their skin conditions had not changed was the same as the number who responded “Increased” (SQ5). In addition, 68.4% of care staff responded that the amount of moisturizing agents used for facility residents increased (SQ8). The answer of “No change” was most common for questions on the number of instructions given to residents (SQ6) and other care staff (SQ7) on methods to apply moisturizing agents; the number of applications of moisturizing agents to residents (SQ9); “Did the rate of transfer of the need to use a moisturizing agent for residents to a nurse change?” (SQ10), “Did the correspondence to a message transferred from a nurse about the skin care change?” (SQ11) and “Did the time required to deal with a skin problem of residents change?” (SQ12).

## Discussion

The results of the questionnaire survey of care staff showed that their understanding of the methods for application of moisturizing agents increased after undergoing just one session of educational training. The frequency of observations of skin conditions of facility residents and the amounts of moisturizing agents used also increased after the training, which suggests that the awareness of skin care increased among the care staff.

In the evaluation of dry skin in residents made by dermatologists, the mean scores for dry skin severity and for scratch marks in the lower extremities decreased significantly (i.e. dry skin improved) from the before to after intervention evaluation. In interviews of residents, the subjective symptoms of dry skin showed a tendency to improve in the after intervention evaluation. The moisturizing function of the skin also improved based on the tendency for increased moisture content in the epidermal stratum corneum, although this change was not significant, and the significant decrease in TEWL from before to after intervention.

There are some limitations in the study. First, the residents were mainly female, and we are uncertain if similar results would be found for elderly male residents. However, there are commonly more female residents in elderly facilities, and it is difficult to adjust the male : female ratio in studies in these facilities. Second, the before and after observations of skin conditions by a dermatologist were limited to those described earlier in the text. More information could have been obtained, but we believe that the observations were detailed enough to provide sufficient data for the analysis in this study. Third, questionnaires completed by residents showed that dermal conditions were improved after intervention, but itching was not changed. Therefore, bias in dry skin between before and after the questionnaire survey cannot be excluded. However, objective measurements by instrument showed improved skin conditions and it is unlikely that the improved conditions were caused only by bias.

Within these limitations, our findings suggest that educational training can improve the understanding of skin care among care staff, and thus improve the skin condition of elderly residents in care facilities. However, there were no changes in the severity of itchiness based on a VRS and in interviews of residents. This suggests the importance of direct visual observation of the skin, because the absence of subjective symptoms of itchiness does not necessarily indicate that there are no skin problems present. This requirement for observation is a particularly important reason for educational training for care staff who work in elderly care facilities.

## Conflict of Interest

Y. T. received fees from Maruho, for research expenses, consultation, lectures and writing. G. N., K. T., N. T., A. K., Y. M., M. O. and H. S. received fees from Maruho for research expenses. M. K. is an employee of Maruho.
